# Editorial: Artificial intelligence technology and the application in medical imaging

**DOI:** 10.3389/fmedt.2022.989983

**Published:** 2022-07-29

**Authors:** Shuaiqi Liu, Yewang Chen, Yu-Dong Zhang

**Affiliations:** ^1^College of Electronic Information Engineering, Hebei University, Baoding, China; ^2^Machine Vision Technology Innovation Center of Hebei Province, Baoding, China; ^3^National Laboratory of Pattern Recognition (NLPR), Institute of Automation, Chinese Academy of Sciences, Beijing, China; ^4^School of Computer Science and Technology, Huaqiao University, Xiamen, China; ^5^School of Computing and Mathematical Sciences, University of Leicester, Leicester, United Kingdom

**Keywords:** artificial intelligence technology, medical imaging, deep learning, disease screening, pathological analysis

In recent years, Artificial Intelligence (AI) has become a research hotspot in academia and industry. It has been successfully applied in the medical and health fields. Artificial intelligence has advantages over human beings in processing big data, complex and uncertain data, and in-depth mining data potential information. Medical data contains rich human health information, which is an important basis for doctors to make medical diagnoses. In the face of complex medical information and the growing demand for medical diagnosis, doctors' artificial interpretation exposes many shortcomings, such as easy to be affected by subjective cognition, low efficiency, and high misdiagnosis rate. With the continuous development of artificial intelligence in the segmentation of lesion area, early diagnosis of disease, anatomical structure, detection of the lesion area, and other aspects, intelligent medical treatment has gradually become possible.

From a technical point of view, medical imaging diagnosis mainly relies on image recognition and deep learning. It is expected that medical image segmentation, feature extraction, quantitative analysis, comparative through artificial intelligence can achieve image diagnosis and treatment. Artificial intelligence can help to interpret medical image data, solve the problems of low diagnostic efficiency and high misdiagnosis rate of doctors to improve the diagnostic efficiency and accuracy and reduce the labor intensity of doctors. In the past few years, major AI medical imaging companies are expanding the business radius with the continuous mature of technology. Breast cancer, stroke, and bone age testing around bone and joint have become the focus of market participants. In novel coronavirus pneumonia, AI medical imaging is involved in quantitative analysis and evaluation of the curative effect of new crown pneumonia, and it has become the key force to improve the diagnostic efficiency and quality of diagnosis.

MRI is a common and widely used medical imaging. Lu et al. proposed a new Cerebral microbleeds (CMB) detection approach based on brain magnetic resonance images. The automated microbleed detection approach combining convolutional neural network (CNN), extreme learning machine (ELM), and bat algorithm (BA) which achieved the-state-of-the-art performance.

Electroencephalogram is another effective non-invasive tool which can directly react to the changes of human brain activity state. An et al. constructs an EEG emotion recognition algorithm based on 3D feature fusion and convolutional autoencoder. Specifically, the differential entropy (DE) features of EEG signals are fused to construct the 3D features of EEG signals. Then, the constructed 3D features are input into the CAE constructed for emotion recognition.

Considering that various modalities of medical imaging are complementary, medical image fusion has an indispensable value. Liu S. et al. proposed a structure preservation-based two-scale multimodal medical image fusion algorithm taking advantage of structure-preserving filter and deep learning.

The above-mentioned articles are based on CNN, however, spiking convolutional neural networks (SCNNs) are energy-saving for sparsity of data flow and event-driven working mechanism compared with CNNs. Chen et al. proposed 4 stopping criterions to reduce the inference-latency of SCNNs during inference phase. The simulation results demonstrated that the stopping criterions can significantly save total inference-latency without obvious accuracy loss. What is more, it provides a new direction for medical image processing.

There are two review papers in this Research Topic. Liu H. et al. elaborate the common steps of an emotion recognition algorithm based on EEG from data acquisition, preprocessing, feature extraction, feature selection to classifier. They also review the existing EEG-based emotional recognition methods and the performances. Luo et al. systematically reviewed the recent literature on segmentation methods for stomatological images based on deep learning and clinical applications. The review shows that DL has great potential and can further promote the transformation of clinical practice from experientialism to digitization, precision, and individuation.

The ultimate goal of this Research Topic is to promote research and development of deep learning for multimodal biomedical images by publishing high-quality research articles, reviews, or perspectives, among other article types, in this rapidly growing interdisciplinary field. We thank the authors of the papers published in this Research Topic for their valuable contributions and the referees for their rigorous review.

## Author contributions

All authors listed have made a substantial, direct, and intellectual contribution to the work and approved it for publication.

## Conflict of interest

The authors declare that the research was conducted in the absence of any commercial or financial relationships that could be construed as a potential conflict of interest.

## Publisher's note

All claims expressed in this article are solely those of the authors and do not necessarily represent those of their affiliated organizations, or those of the publisher, the editors and the reviewers. Any product that may be evaluated in this article, or claim that may be made by its manufacturer, is not guaranteed or endorsed by the publisher.

